# The paternal genetic legacy of Hungarian-speaking Rétköz (Hungary) and Váh valley (Slovakia) populations

**DOI:** 10.3389/fgene.2022.977517

**Published:** 2022-10-17

**Authors:** Horolma Pamjav, Ábel Fóthi, Dániel Dudás, Attila Tapasztó, Virág Krizsik, Erzsébet Fóthi

**Affiliations:** ^1^ Department of Reference sample analysis, Institute of Forensic Genetics, Hungarian Institutes for Forensic Sciences, Budapest, Hungary; ^2^ Institute of Archaeogenomics, Research Centre for the Humanities, Budapest, Hungary; ^3^ Departmant of Genetics, Eötvös Lorand University, Budapest, Hungary

**Keywords:** Y haplotypes and haplogroups, Hungarian-speakers, Rétköz and Váh valley populations, human demographic history, Y-STRs and Y-SNPs

## Abstract

One hundred and six Rétköz and 48 Váh valley samples were collected from the contact zones of Hungarian-Slovakian territories and were genotyped for Y-chromosomal haplotypes and haplogroups. The results were compared with contemporary and archaic data from published sources. The genetic composition of the Rétköz population from Hungary and the Váh valley population from Slovakia indicates different histories. In the Rétköz population, the paternal lineages that were also found in the Hungarian Conquerors, such as R1a-Z93, N-M46, Q-M242, and R1b-L23, were better preserved. These haplogroups occurred in 10% of the population. The population of the Váh valley, however, is characterized by the complete absence of these haplogroups. Our study did not detect a genetic link between the Váh valley population and the Hungarian Conquerors; the genetic composition of the Váh valley population is similar to that of the surrounding Indo-European populations. The Hungarian Rétköz males shared common haplotypes with ancient Xiongnu, ancient Avar, Caucasian Avar, Abkhazian, Balkarian, and Circassian males within haplogroups R1a-Z93, N1c-M46, and R1b-L23, indicating a common genetic footprint. Another difference between the two studied Hungarian populations can be concluded from the Fst-based MDS plot. The Váh valley, in the western part of the Hungarian-Slovakian contact zone, is genetically closer to the Western Europeans. In contrast, Rétköz is in the eastern part of that zone and therefore closer to the Eastern Europeans.

## 1 Introduction

The Carpathian Basin was historically the destination for several nomadic tribes that migrated westwards from Inner and Central Asia towards Europe. The ancient Hungarians (Steppe Magyars) entered the Carpathian Basin from the east in the ninth century CE and settled there (Róna-Tas, 1999). The genetic legacy of the populations in the Carpathian Basin can be studied only if the genetic structure of them is compared to that of ancient populations. Fortunately, in recent years, the number of aDNA genetic results based on both uniparental and whole genome data has increased in Hungary (Neparáczki et al., 2017a; Neparáczki et al., 2017b; Neparáczki et al., 2018; Olasz et al., 2018; Neparáczki et al., 2019; Csáky et al., 2020; Fóthi et al., 2020; Maár et al., 2021; Nagy et al., 2021). Several Y-STR and Y-SNP results from contemporary Hungarian-speaking populations have been published (Völgyi et al., 2009; Bíró et al., 2015; Pamjav et al., 2017). However, fewer genetic results exist from populations living in geographically isolated areas in the Carpathian Basin, such as the Bodrogköz (Pamjav et al., 2017).

Based on published uniparental genetic data, the contemporary Hungarians possess some genetic similarities to ancient conquerors in the Carpathian Basin, as well as to Central/Inner Asian populations and populations in the Ural Mountains (Bíró et al., 2015; Fehér et al., 2015; Neparáczki et al., 2017a; Pamjav et al., 2017; Huang et al., 2018; Dudás et al., 2019; Csáky et al., 2020). The core of the Hungarian Conquerors may have originated from Inner Asia/Southern Siberia, and then likely admixed with the peoples they encountered during their migration westwards. Examples of this may be found in the Ural and the Caucasus Mountains. The Y-chromosomal relationship for Inner Asian origin is the presence of haplogroups C-M86, R1a-Z93, and N-M46 in ancient or present-day Hungarian populations (Pamjav et al., 2017; Fóthi et al., 2020). The Y-chromosomal relationship for Ural or Caucasian origin is the presence of Y-haplogroups N-L1034, R1b-L23, Q-M242, and G2a-L156 in Hungarian conquerors or modern Hungarian populations (Biró et al., 2015; Pamjav et al., 2017; Fóthi et al., 2020). The mtDNA relationship for Inner Asia/Southern Siberia origin is the presence of mtDNA haplogroups A, B, H6, and T1a* in the Hungarian Conquerors or present-day Hungarians (Neparáczki et al., 2017a). The mtDNA haplogroup X2f represents a genetic link to Caucasian populations (Neparáczki et al., 2017a). Other mtDNA studies of early Hungarians revealed a diverse composition of haplogroups with significant Asian affinity (Bogács-Szabó et al., 2005; Branstatter et al., 2007; Tömöry et al., 2007) and concluded that the influence of Asian migration into Eastern Europe is detectable in the Székely (or Sekler) population (Romania) and the relatively high proportion of Asian mtDNA haplogroups A, B, C, G, and Y, totaling 7.9% altogether, may be an indicate an influx of Asian nomads into the Székely population in medieval Transylvania (Branstatter et al., 2007).

Thus, the present-day Hungarian speakers have a very complex genetic history. Moreover, the population consists of several ethnic groups in the Carpathian Basin, such as the Székely, Csángó, Palóc, Jász, and Kun. However, Y-chromosomal studies about them and other regional population groups are still limited.

In our previous study, we studied paternal genetic composition of the Bodrogköz population in Eastern Hungary and found genetic similarities to that of ancient Hungarians (Pamjav et al., 2017). To continue the study, we analyzed two Hungarian speaking populations, the Rétköz population in Eastern Hungary, as well as the Váh valley population in Slovakia. To explore their population histories, all Rétköz and Váh valley samples were surveyed for 23 Y-STRs and over 40 Y-SNPs. The resulting data were compared to our published Y-chromosomal data from Eurasian populations and contemporary Hungarians, as well as to Eurasian populations from other published studies.

The Rétköz is a region in Eastern Hungary, whereas the Váh valley is in Western Slovakia. The populations of both places live within the Hungarian-Slovakian contact zone and are Hungarian-speakers. The Rétköz is near the Bodrogköz (Pamjav et al., 2017), but genetic studies have not been conducted in this region or in the Váh valley. The common features of these three regions (Bodrogköz, Rétköz and Váh valley) are that ancient Hungarian Conquerors lived there and that many of their cemeteries have been excavated.

In this study, we present new Y-STR and Y-SNP data of two Hungarian-speaking populations in the contact zone of Hungarian-Slovakian territories and compare them to contemporary Eurasian and available aDNA data to gain further knowledge about the genetic history of these populations.

## 2 Materials and methods

### 2.1 Materials

**FIGURE 1:**
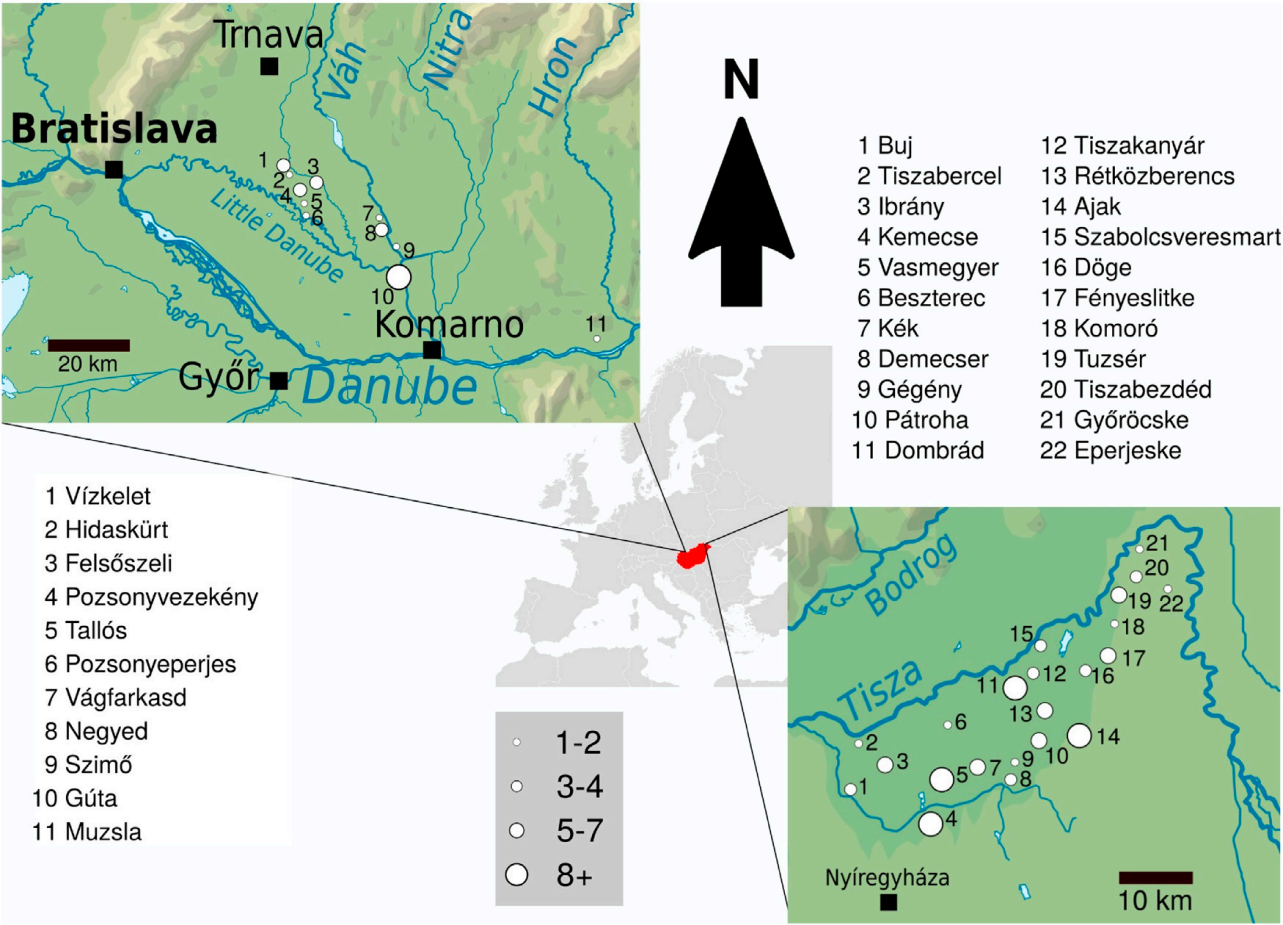
The geographical distribution of 48 and 106 non-related Hungarian males from Váh valley and Rétköz, respectively. Left side: Map and list of the sampled Váh valley settlements. Right side: Map and list of the sampled Rétköz settlements. Settlements are marked with circles of different sizes that are proportional to the number of individuals sampled.

We collected samples from 106 unrelated males from Rétköz, Hungary and 48 males from the Váh valley, Slovakia (Figure 1). Informed consent was obtained from all live participants included in the study.

### 2.2 Methods

#### 2.2.1 Testing of Y-STR and Y-SNP markers

Genomic DNA was extracted from buccal swabs using the Investigator kit and EZ1 robotic system (Qiagen, Germany), as described in the manufacturer’s instructions. The samples were quantified using the Quantifiler Human kit and the ABI 7500 Real-time PCR System (Thermo Fisher Scientific, Waltham, MA, USA).

DNAfrom the Rétköz and Váh valley populations was surveyed for genetic variation using the Promega PowerPlex Y23 kit. Allele sizing and calling were determined with the ABI3500 Genetic Analyzer and GeneMapper ID-X v.1.4 software. To test for Y-SNP markers (Y chromosomal Single Nucleotide Polymorphism), we performed amplifications of 1–2 ng genomic DNA with Custom TaqMan probes and analyzed the relative fluorescence of the PCR productsin an ABI 7500 Real-time PCR instrument using SDS.1.2.3 software. The SNP markers tested were CT-M168, KT-M9, PR-M45, T-M170, I-M170, I1-M253, I2b-M223, I2a-P37, J-M304, J1-M267, J2-M172, J2-M67, J2b-M12, R-M207, R1-M173, R2-M124, R1a-M198, R1a-SRY1083.1, R1a-M458, R1a-Z93, R1a-Z280, R1b-M343, R1b-P25, R1b-U106, R1b-P312, R1b-M412, R1b-Z2103, D-M174, N-M231, N1-LLy22g, N-L708, N-M46, N-L1034, N-VL29, N-Z1936, N-F4205, R1a-Y2633, and N-Y24365. The haplogroups are described in accordance with the generally accepted nomenclature, as is common practice (Karafet et al., 2008 and the ISOGG).

The Y-STR (Y chromosomal Short Tandem Repeat) haplotypes in this study were sent to the YHRD (accession numbers: YA004754 for the Rétköz and YA004755 for the Váh valley populations).

#### 2.2.2 Phylogenetic analysis

To examine the STR variation within the haplogroups, relational networks were constructed using the Network 10.2.0.0 program (Bandelt et al., 1999). Repeats of the DYS389I locus were subtracted from the DYS389II locus, and the DYS385 locus was excluded because the Network program cannot handle the duplicated locus. To put the results into a more extensive geographical context, we included haplotypes of 10 overlapping evolutionarily stabile loci from other Eurasian populations. The rho statistic in the network program was used to estimate the time to the most recent common ancestor (TMRCA) of haplotypes among the compared haplogroups (Bandelt et al., 1999). Evolutionary time estimates were calculated according to Zhivotovsky et al. and STR mutation rate was assumed to be 6.9 × 10^−4^/locus/25 years (Zhivotovsky et al., 2004) as is common practice. STR-based TMRCA estimates were for information only and are not discussed in this paper due to their unreliability and insignificance to the primary purpose of the study.

#### 2.2.3 Genetic structure

Based on the Y-STR haplotypes, pairwise Rst (stepwise mutation) genetic distances were computed with YHRD. org’s online AMOVA, and the MDS plot constructed (release 66) (Willuweit and Roewer 2015). Pairwise Fst genetic distances were calculated based on haplogroup frequencies using Arlequin 3.5 software (Excoffier and Lischer, 2010). MDS plot from the Fst values was constructed with the cmdscale function of R (R Core Team, 2021). Rst is an analogy of Fst based on allele size differences; it is defined as the correlation of allele sizes between tested markers within populations. Fst (fixation index) is more efficient when there are high levels of gene flow, whereas Rst reflects population differentiation better under low gene flow (Balloux and Goudet, 2002). That’s why we used both Rst and Fst-based metric MDS analyses for population comparisons.

Haplotype and haplogroup frequencies and their diversity values were calculated using the formula from Nei 1973.

## 3 Results

### 3.1 Y-chromosome diversity

The haplogroup frequencies of the two populations from the Hungarian-Slovakian contact zone are presented in Table 1. The STR and SNP results of the 106 Rétköz and 48 Váh valley males are shown in Supplementary Table S1. The most frequent haplogroups of the Rétköz population were R1a-Z280 (19.8%), R1a-M458 (17%), R1b-P312 (12.3%), R1b-P25/M343 (7.55%), E1b1-M78 (6.6%), I2a-p37 (6.6%), G2a-L156 (6.6%), and I1-M253 (4.7%). Furthermore, haplogroups J2b-M12, Q-M242, and R1b-U106 accounted for 2.83% of each haplogroup. I2b-M223 was 1.89% of the Rétköz population, whereas the remaining haplogroups, including the Rétköz N-M46 (earlier N1c) chromosomes, which belong to the studied subgroups (N-L1034, N-VL29, N-Z1936), accounted for less than 1% of the lineages.

**TABLE 1:** Haplogroup frequencies of the Rétköz and Váh valley populatios studied.

Haplogroups	Rétköz	%	Váh valley	%	N	%
**E1b1-M123**	1	0.94	2	4.17	3	1.95
**E1b1-M78**	7	6.60	7	14.58	14	9.09
**G2a-L156**	7	6.60	2	4.17	9	5.84
**H1a-M82**	1	0.94	0	0.00	1	0.65
**I1-M253**	5	4.70	2	4.17	7	4.55
**I2a-P37**	7	6.60	8	16.67	15	9.74
**I2b-M223**	2	1.89	1	2.08	3	1.95
**J2a-M67**	1	0.94	2	4.17	3	1.95
**J2b-M12**	3	2.83	0	0.00	3	1.95
J2*-M172	0	0.00	1	2.08	1	0.65
**N1c-L1034**	1	0.94	0	0.00	1	0.65
**N1c-VL29**	1	0.94	0	0.00	1	0.65
**N1c-Z1936**	1	0.94	0	0.00	1	0.65
**Q-M242**	3	2.83	0	0.00	3	1.95
R1a*-M198	0	0.00	1	2.08	1	0.65
**R1a-M458**	18	17.00	3	6.25	21	13.64
R1a*-SRY10831	1	0.94	0	0.00	1	0.65
**R1a-Z280**	21	19.80	8	16.67	29	18.83
**R1a-Z93**	2	1.89	0	0.00	2	1.30
R1b*-M343/P25	8	7.55	3	6.25	11	7.14
**R1b-P312**	13	12.30	1	2.08	14	9.09
**R1b-U106**	3	2.83	7	14.58	10	6.48
**22**	106	100.00	48	100.00	154	100.00

In the case of the Váh valley males, the most frequent haplogroups were I2a-P37 and R1a-Z280 (both at 16.67%). The frequencies of the remaining haplogroups were as follows: E1b1-M78 (14.58%), R1a-M458 (6.25%), R1b-P25/M343 (6.25%), E1b1-M123 (4.17%), G2a-L156 (4.17%), I1-M253 (4.17%), J2a1-M67 (4.17%), and the remaining haplogroups (2.08%). However, nearly 10% of the present-day Rétköz population may be related to the ancient Hungarians based on haplogroup composition, but the modern Hungarians in the Váh valley lack such relatedness.

The haplotype and haplogroup diversities of the Rétköz group were 1.00 and 0.901, respectively, whereas these values for the Váh valley were 0.998 and 0.904, respectively. The results show that, in both populations, haplotypes are more diverse than haplogroups.

### 3.2 Phylogenetic analysis

Based on the Y-STR haplotypes, networks were constructed for haplogroups that may be linked to those of the Hungarian Conquerors, because these haplogroups can be found in ancient Hungarians and originated from Inner/Central Asia. We also included 10 Y-STR haplotypes from other Eurasian populations and aDNA results from published sources to widen the geographical context. We have included six networks (R1a-Z93, N-Tat, Q-M242, R1b-P25/M343, E1b1-M78, and G2a-L156) that are potentially helpful in uncovering the genetic legacy of the populations being studied. Other networks, which were constructed for haplogroups of European origin (R1a-M458, R1a-Z280, R1b-P312, R1b-U106 and I2a-P37) and could not be related to Hungarian Conquerors, were not included in the study. Supplementary Table S2 summarizes data for all the published aDNA samples used in the study including haplotypes, haplogroups, ages and geographic origins.

#### 3.2.1 Median joining network of 121 R1a-Z93 haplotypes

**FIGURE 2:**
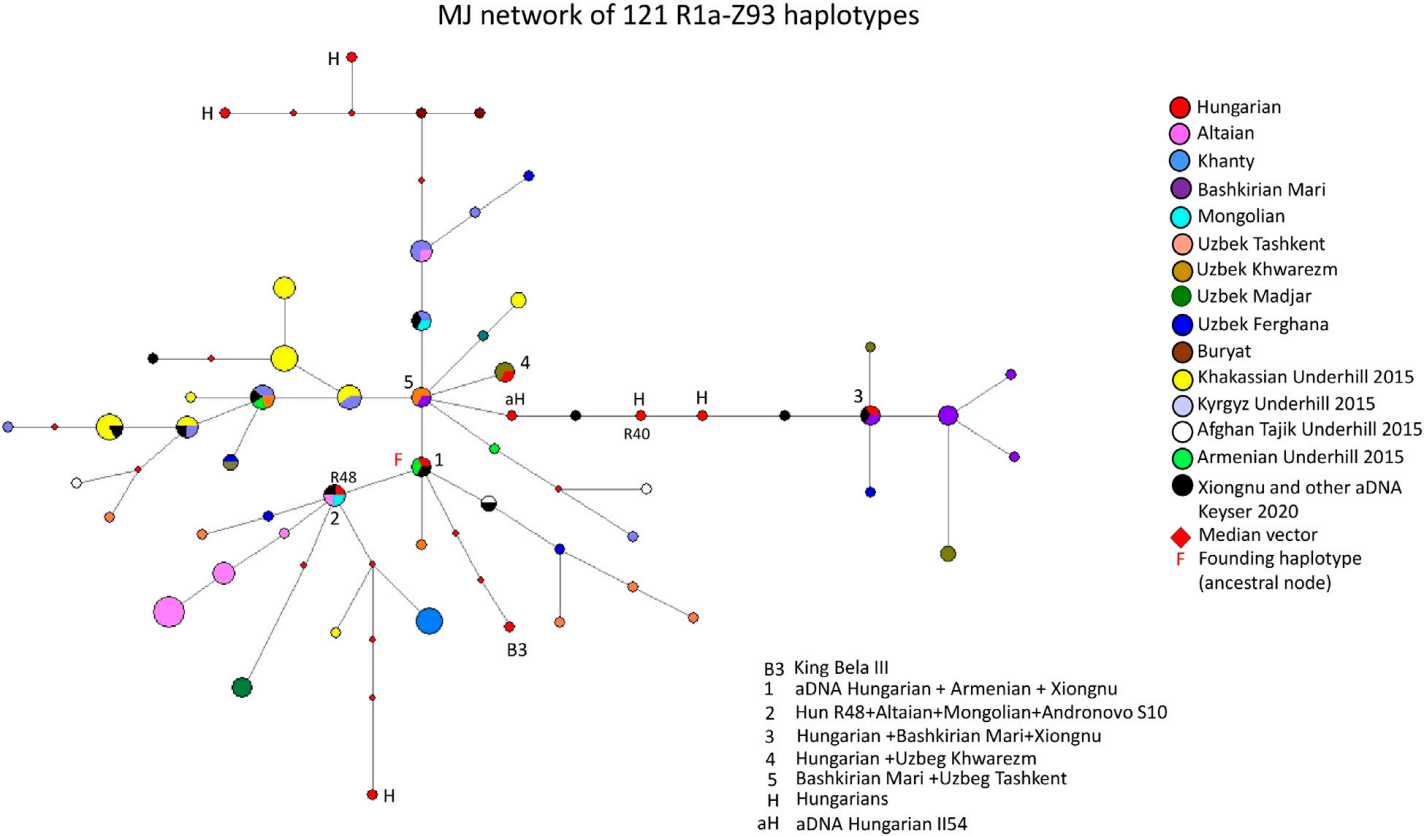
The Median-Joining Networks (MJ) of 121 R1a-Z93 haplotypes. The circle sizes are proportional to the haplotype frequencies. The smallest area is equivalent to one individual.

Figure 2 depicts an MJ network of 121 R1a-Z93 haplotypes from the 15 populations tested by us for this study or previously published (Bíró et al., 2015; Underhill et al., 2015; Dudás et al., 2019) and aDNA samples (Olasz et al., 2018; Fóthi et al., 2020; Keyser et al., 2021). Three modern Hungarian and one aDNA samples (II54 from the Hungarian Royal Basilica of Székesfehérvár), including 1 Rétköz sample (Rétköz 40), formed a common branch with three ancient Xiongnu samples (TUK45, TUK04, TUK25) on the right side of Figure 2. On this branch, one modern Hungarian sample shared two haplotypes (1 TUK25 and one Bashkirian Mari) in haplotype cluster 3. Two Uzbek samples (1 Khwarizm and one Fergana sample) can be derived from cluster 3 (see Figure 2). The five Bashkirian Mari and two Uzbek (Khwarizm) samples are clustered one molecular step from cluster three at the end of this branch. Cluster five includes one Bashkirian Mari and two Uzbek males from Tashkent in Uzbekistan and located at one molecular step from cluster 1. Cluster one includes three haplotypes: one Hungarian aDNA (Nagykörös Gr2), one Xiongnu aDNA (TUK09A) and one modern Armenian sample, separated by one molecular step (DYS389I) from cluster 5. The founding haplotype, which may have arisen in the common ancestor of populations or males and is shared by them. Based on this, cluster 1 may be the founding haplotype, as it contains two ancient haplotypes from males that lived 1,000 to 2,500 years ago. All the samples and branches are derived from this haplotype. Cluster two includes one Rétköz Hungarian (Rétköz = R48), one Mongolian, one Altaian, and one Andronovo aDNA (S10) samples. Cluster four can be separated one molecular step from cluster five and includes one Hungarian and two Uzbek males from Tashkent in Uzbekistan. The Hungarian King Bela III is found three molecular steps away from cluster 1 (see B3 in Figure 2). The remaining three Hungarian haplotypes are outliers in the network and are not shared by any of the samples.

The other studied samples in the network either form independent clusters, such as Altaians, Khakassians, Khanties, and Uzbek Madjars, or are scattered within the network. Other aDNA samples (S26, ARZT1, ARZT28, and MN376 from Keyser et al., 2021) in the network form a different branch, with almost all Khakassian samples located on the left side of Figure 2. The age of accumulated STR variation (TMRCA = Time to Most Recent Common Ancestor) within the R1a-Z93* lineage for 121 samples is estimated as 13 ± 3 kya (95% CI = 10.0–16.0 kya), considering that cluster one is the founder haplotype, which is higher than that of the SNP-based calculation (4.6 kya, 95% CI: 4.2–5.0 kya) (Adamov et al., 2015; www.yfull.com).

#### 3.2.2 Median-joining network of 179 N-M46 haplotypes

**FIGURE 3:**
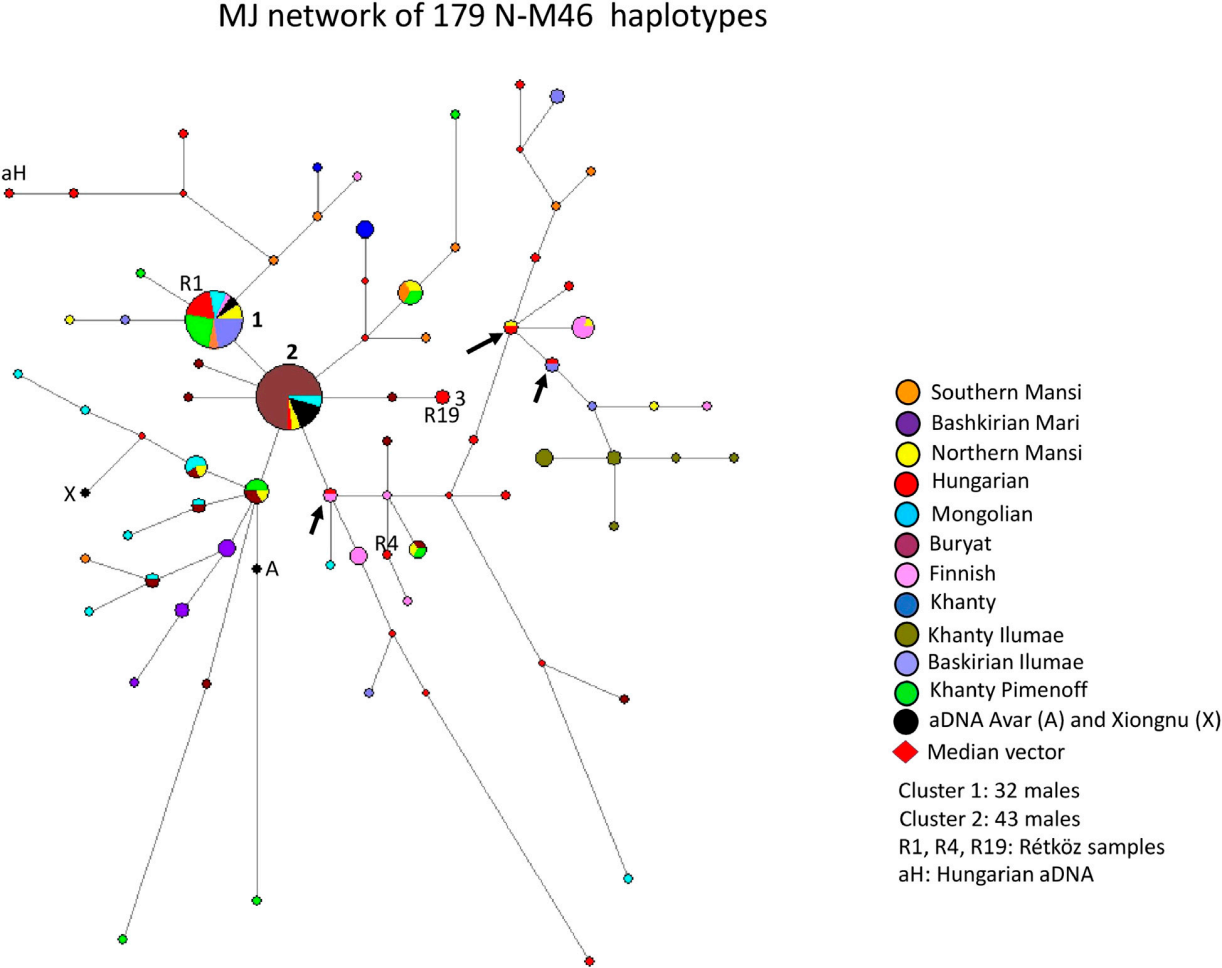
The Median-Joining Networks (MJ) of 179 N-M46 haplotypes. The circle sizes are proportional to the haplotype frequencies. The smallest area is equivalent to one individual.

A median-joining network of 179 N-M46 (previously N1c, Karafet et al., 2008) haplotypes was generated using populations we previously studied (Bíró et al., 2015; Fehér et al., 2015), as well as those researched by others (Pimenoff et al., 2008; Ilumäe et al., 2016) (Figure 3). The founder N-M46 (Tat = M46) haplotype was shared by 32 samples from eight populations (8 Khanty from Pimenoff et al., 2008; three Northern Mansi; three Mongolian; six Hungarian-speakers, including the Rétköz 01 sample; two Southern Mansi; seven Bashkirian from Ilumäe et al., 2016; two ancient Avars from Csáky et al., 2020, and one Finnish), as seen in Figure 3 (haplotype cluster 1). Cluster one is the founding haplotype. Cluster 2, another large cluster, includes 43 haplotypes (32 Buryat; two Mongolian; one Hungarian; two Northern Mansi; and six ancient Avars from Csáky et al., 2020) from five populations, as shown in Figure 3. Cluster two is located one molecular step from cluster 1. The only difference is in the DYS391 locus, with allele 11 in cluster one and allele 10 in cluster 2. As seen in Figure 3, 73% of Buryats belonged to cluster 2, indicating that the Buryats we studied belong to a young and isolated population (Dudás et al., 2019). Two Hungarian males, including Rétköz 19 (R19) sample, derives from cluster two *via* one Buryat haplotype (see three in Figure 3). One Hungarian aDNA haplotype (Ö52/50, Fóthi et al., 2020) was positioned six mutational steps away from cluster one and formed a haplotype branch with two present-day Hungarian males (see aH in Figure 3). Three modern Hungarian males shared 1-1 haplotypes with Finnish, Northern Mansi, or Bashkirian males (see black arrows in Figure 3).

The other population samples included in the network formed independent clusters, such as Bashkirian Mari, Finns, Khanties, Bashkirians, and Khanties from another research (Ilumäe et al. (2016). They also shared haplotype clusters or were scattered in the network.

The age of accumulated STR variation (TMRCA) within N-M46 lineage for 179 samples is estimated as 11.3 ± 3.3 kya (95% CI = 8.0–14.6 kya), considering cluster one is the founder haplotype, which is similar to its sequence-based calculation of 13 kya (95% CI:11.3–14.6 kya) (Ilumäe et al., 2016).

#### 3.2.3 Median-joining network of 153 R1b-M343 haplotypes

**FIGURE 4:**
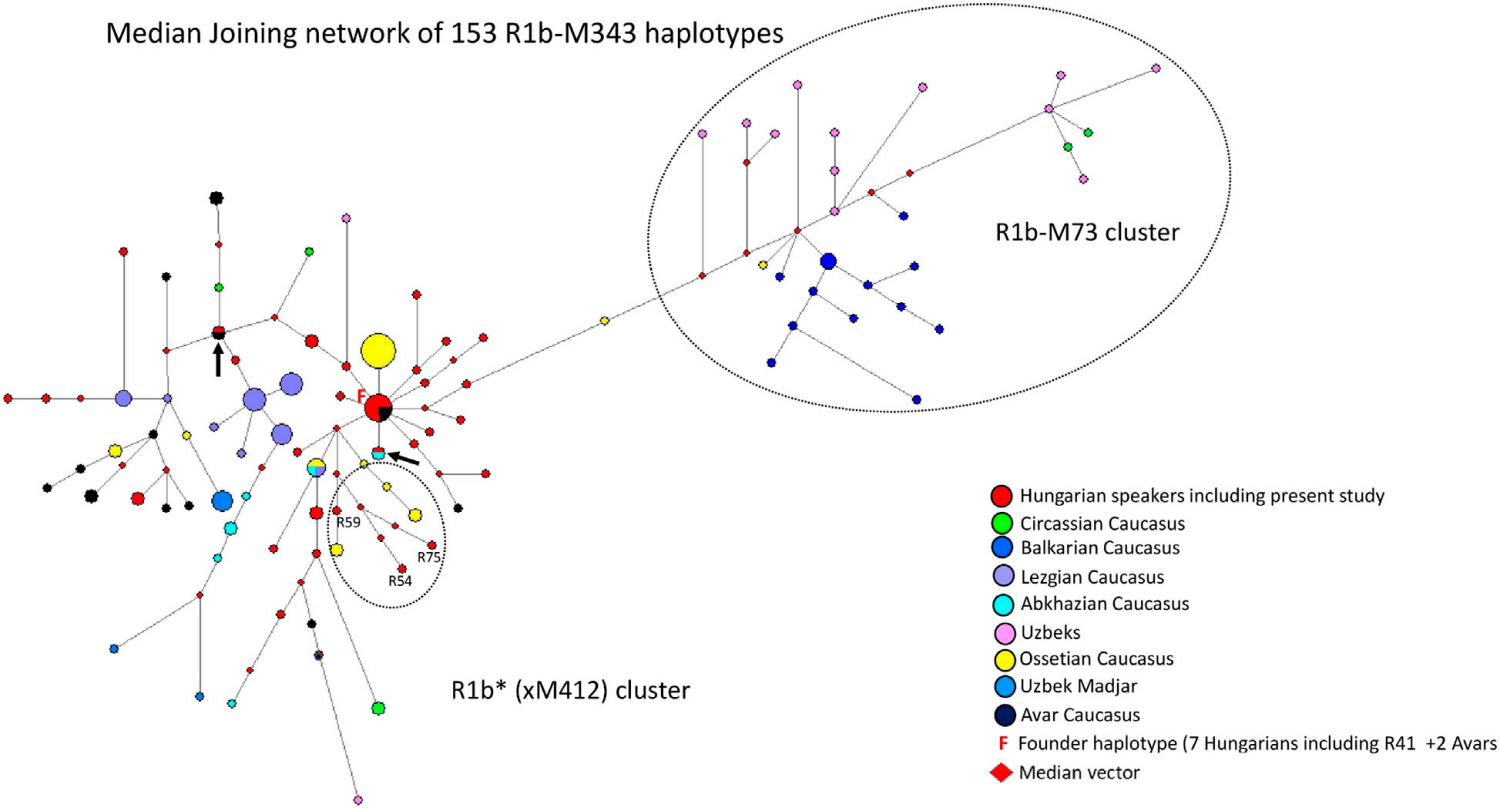
The Median-Joining Networks (MJ) of 153 R1b-M343* (P25) haplotypes. The circle sizes are proportional to the haplotype frequencies. The smallest area is equivalent to one individual.

A median-joining network of 153 R1b-M343/P25 (Eastern R1b-L23) haplotypes was generated using samples from FTDNA, populations we previously studied (Balanovsky et al., 2011; Bíró et al., 2015) (Figure 4). The founder R1b*-M343 (L23) haplotype was shared by nine samples, including the Rétköz 41 (R41) sample, from two populations (7 Hungarians and two Avars from the Caucasus), as shown in Figure 4. The majority Hungarian haplotypes, including four Rétköz (R28, R52, R39 and R37) and one Váh valley samples (V24), appear to be descended from the founding haplotype (F = founder), because these haplotypes differ one molecular step from the founding haplotype. The pattern of these haplotype clusters is starlike, representing a set of closely related haplotypes of Hungarian males. Three Rétköz R1b samples (R54, R59 and R75) clustered with Ossetian samples from the Caucasus (indicated by the red circles in a dashed circle under the founding haplotype cluster in Figure 4.). In addition to the founding haplotype, some Hungarian haplotypes were shared with Abkhazian (turquoise and red circles) or with Hungarians and Avars from the Caucasus (red and black circles), which are marked with an arrow. All other Hungarian haplotypes were scattered within the network. It is interesting that either Hungarian haplotypes shared a common haplotype with Avar males or clustered with Avar haplotypes in the network (red and black circles).

The other population samples in the network formed independent clusters, such as Lezgian, Ossetian, and Circassian from the Caucasus or were scattered in the network. Primarily Balkarian (Caucasus) and Uzbek males form an independent branch (R1b-M73) on the right side of the network (Figure 4). The age of accumulated STR variation within the R1b-M343/P25 lineage for 153 samples is estimated to be 22.8 ± 3.8 kya (95% CI = 19.0–26.6 kya), considering the founder haplotype is the ancestral one (F in Figure 4), which is in agreement with the time calculated on sequence data (yfull.com). For the R1b (xM412) branch, the age of accumulated STR variation is 17.7 ± 5.2 kya (95% CI = 12.5–22.9 kya), which is much higher than whtat Myres et al., 2011 calculated.

#### 3.2.4 Median-joining network of 167 Q-M242 haplotypes

**FIGURE 5:**
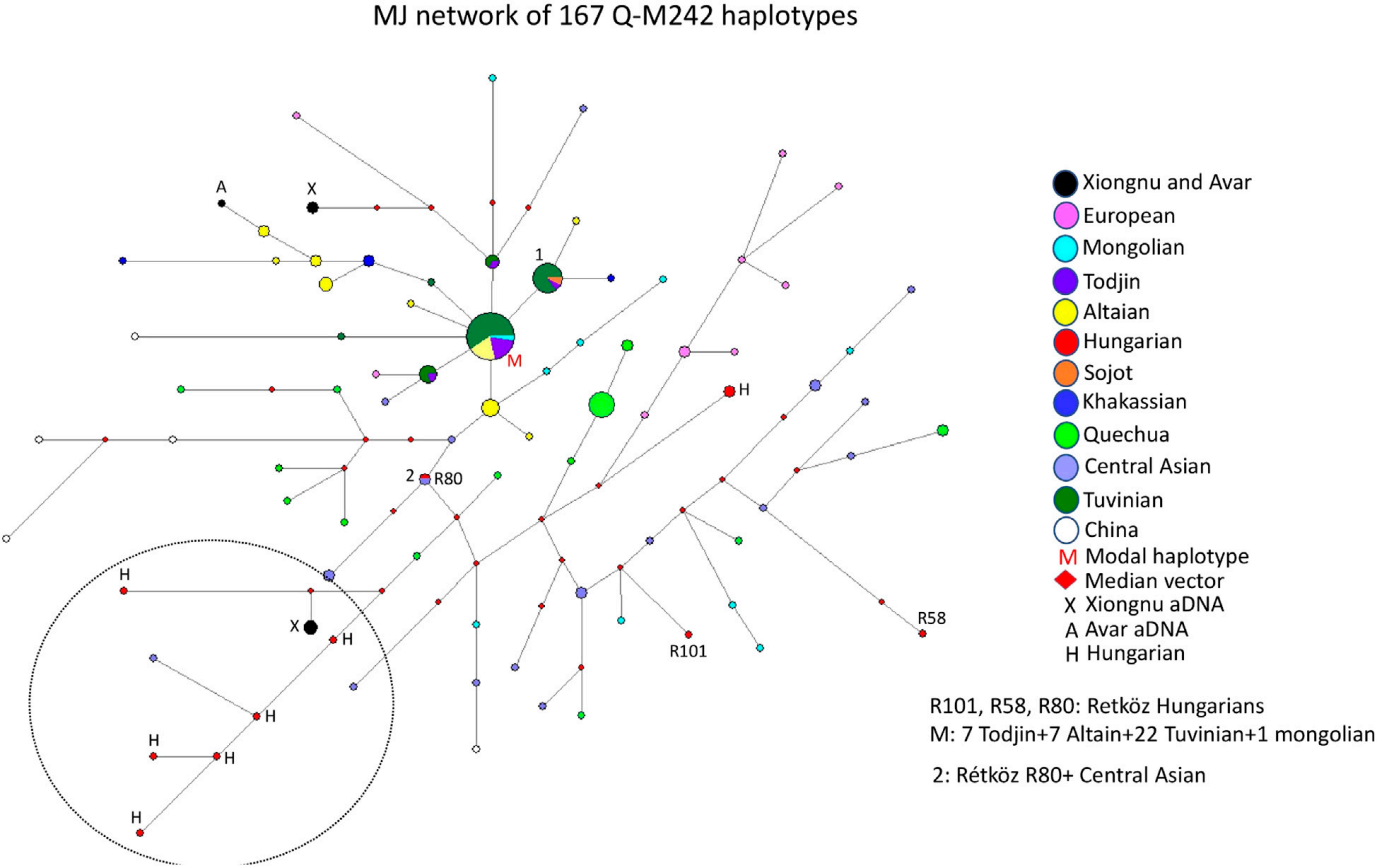
The Median-Joining Networks (MJ) of 167 Q-M242 haplotypes. The circle sizes are proportional to the haplotype frequencies. The smallest area is equivalent to one individual.

The median-joining network of 167 Q-M242 haplotypes from 12 populations does not show a star-like pattern, but rather a more diverse one. However, it was simple to analyze the genetic relationship of the Hungarian samples (Figure 5). The largest haplotype cluster, to which a relatively large number of individuals belong, is shared by four populations (see M on Figure 5). This cluster includes 22 Tuvinian, seven Todjin, seven Altaian, and one Mongolian haplotypes. Another large haplotype cluster (cluster 1) includes 14 males from three populations (2 Sojot, one Tojin, and 11 Tuvinian males), whereas almost all other haplotypes are scattered within the network.

The Rétköz 80 (R80) sample shares the same haplotype as an Uzbek male, and the Rétköz 101 (R101) sample clusters with a Mongolian male. The third Rétköz male (R58) is located six steps away from an Uzbek sample and is an outlier.

It is noteworthy that some Hungarian samples, primarily Hungarian-speaking Csángó and Székely males in Transylvania, Romania form a separate branch with the ancient Xiongnu males (TUK01, TUK20, and TUK26) (see the black and red circles within the dashed circle in the lower left part of Figure 5), indicating a close genetic affinity with each other. Two ancient Xiongnu (TUK43 and TUK44) samples and one ancient Avar sample from published sources (Csáky et al., 2020; Keyser et al., 2021) are more closely related to a European (Lithuanian) and an Altai sample (see the top of Figure 5.), which indicate different histories. We constructed another network, including Jewish and Balkarian samples, with 192 haplotypes. Two Hungarian males (Hu423 and E699) formed a cluster with the Jewish samples, suggesting a genetic link (data not shown). These Hungarian samples form a cluster with European samples on the right side of Figure 5 (see red H and pink circles). Three Hungarian samples (Csangó 68, Székely 180, and Hu1769) form a branch with Balkarian males (data not shown). These three Hungarian samples form a branch with other Hungarian and Xiongnu samples in Figure 5. Based on this network, we have distinguished the Hungarian samples that have a genetic relationship with the Jewish and Balkarian samples.

The age of accumulated STR variation within the Q-M242 lineage for 167 samples is estimated to be 17.7 ± 3.6 kya (95% CI = 14.1–21.3 kya), considering that the modal haplotype (Figure 5) is the founder, which agrees with the previous calculated 15–25 ky (Karafet et al. (2008).

#### 3.2.5 Median-joining network of 189 G2a-L156 haplotypes

**FIGURE 6:**
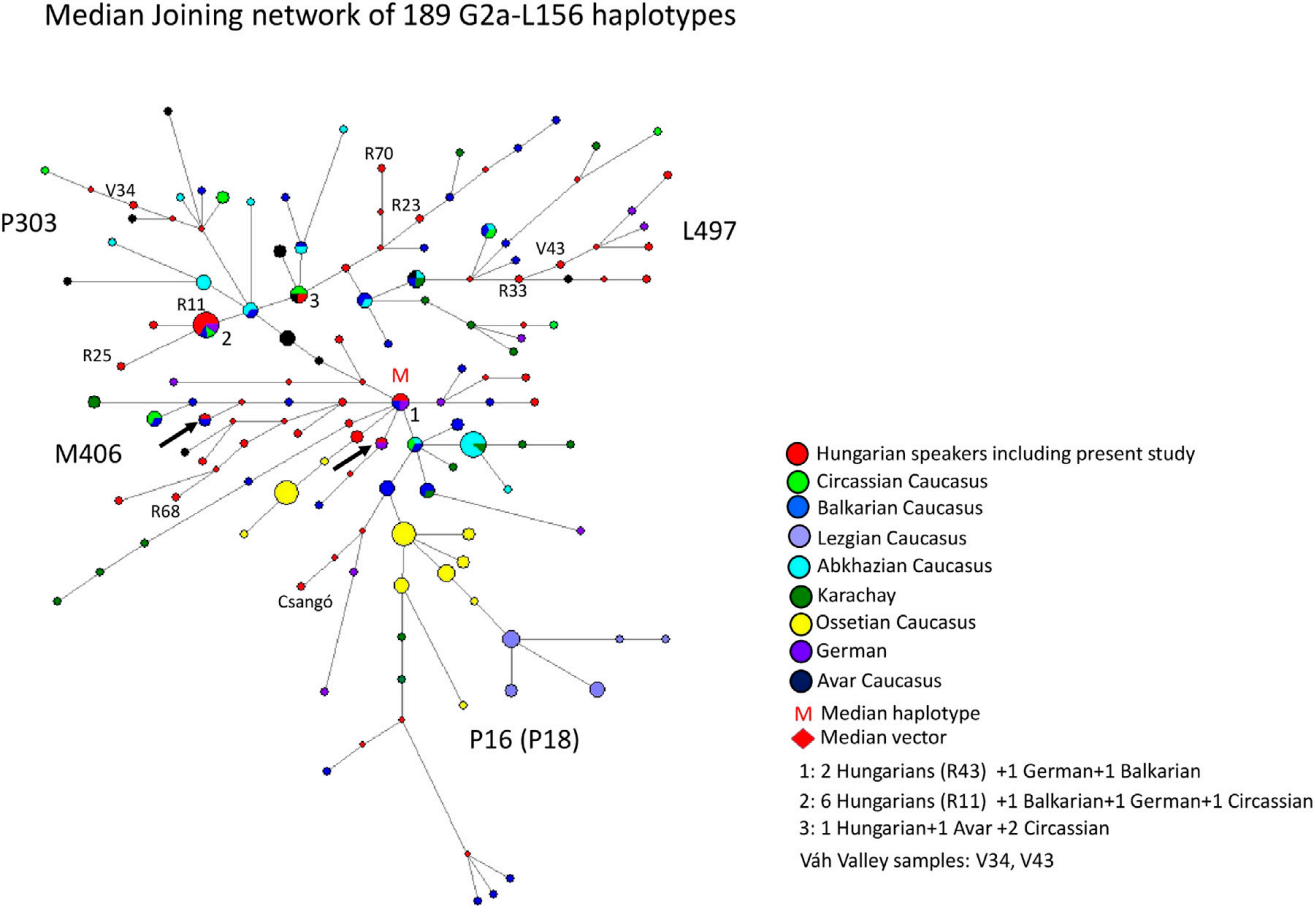
The Median-Joining Networks (MJ) of 189 G2a-L156 haplotypes. The circle sizes are proportional to the haplotype frequencies. The smallest area is equivalent to one individual.

The MJ network of 189 G2a-L156 haplotypes is depicted in Figure 6. The samples belong to four subgroups (P303, L497, M406 and P16). The model haplotype cluster (cluster 1 = M in Figure 6) is shared by three populations, including two Hungarian, one German and one Balkarian males. The haplotype cluster is likely to be the founder haplotype, as all branches are derived from this and is the median center of the branches. The biggest haplotype cluster is shared by four populations, including six Hungarians (Rétköz 11 = R11), one German, one Balkarian, and one Circassian chromosome fromthe Caucasus (cluster two in Figure 6). Cluster three is shared by three populations, including one Hungarian, one Avar and two Circassian males from the Caucasus. One Hungarian male matches a German haplotype (red-purple circle), and another Hungarian male matches with a Balkarian male (red-dark blue circle in Figure 6), indicating a common origin with Hungarians. We marked these haplotypes with an arrow in Figure 6, as well. All other Hungarian males derive from clusters 1-3, and a shared haplotype was not observed. It appears that the Hungarian haplotypes, including Rétköz (R11, R23, R33, R68 and R70) and Váh valley (V34, V43) samples, are generally clustered together on a common branch with Balkarian/Karachay, Avar, or German haplotypes, suggesting that these chromosomes have common origin. Ossetian, Lezgian, and Abkhazian males from the Caucasus form independent clusters, except for the Circassians, which are scattered within the network.

The age of accumulated STR variation within the G2a1-L156 lineage for 189 samples is estimated to be 19.2 ± 3.7 kya (95% CI = 15.5–22.9 kya), considering the modal haplotype (Figure 6) is the founder one. This is in line with the 15.0 ky calculated by Rootsi et al. (2012).

#### 3.2.6 Median-joining network of 183 E1b1-M78 haplotypes

**FIGURE 7:**
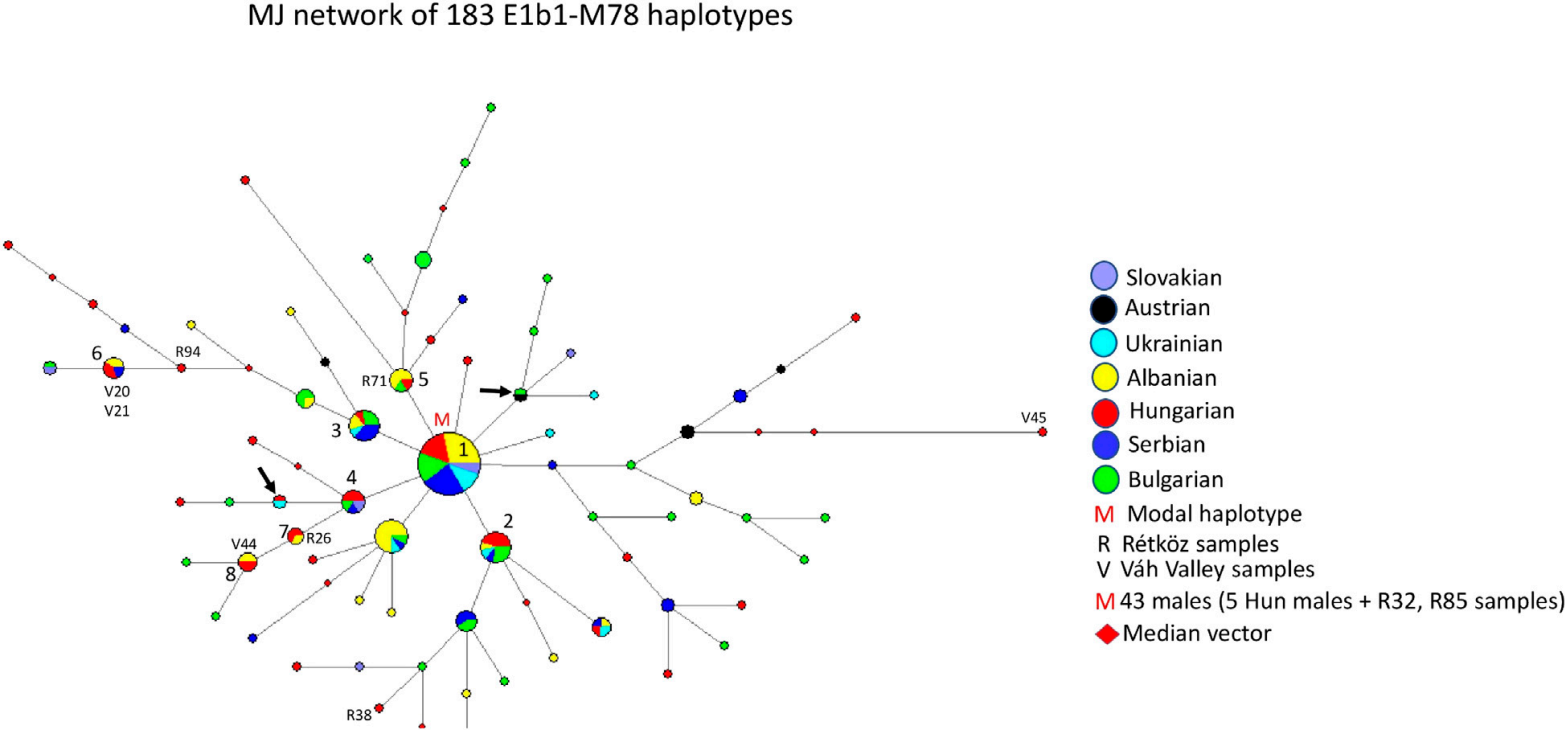
The Median-Joining Networks (MJ) of 183 E1b1-M78 haplotypes. The circle sizes are proportional to the haplotype frequencies. The smallest area is equivalent to one individual.

Figure 7 depicts the median-joining network of 183 E1b1-M78 haplotypes. The network shows a star-like pattern. The biggest cluster (cluster 1 = M) is the modal haplotype shared by six populations, including 43 males. The model haplotype includes seven Hungarian (R32 and R85), 12 Albanian, five Ukrainian, 10 Serbian, seven Bulgarian, and two Slavic haplotypes. There are several haplotype clusters showing different admixture of the seven populations researched in the study, such as cluster 2 (5 Hungarian, one Albanian, one Ukrainian, one Serbian, and three Bulgarian males), cluster 3 (1 Hungarian, two Albanian, one Ukrainian, four Serbian, and three Bulgarian males), cluster 4 (3 Hungarian, one Bulgarian, one Serbian, and one Slavic males), cluster 5 (4 Albanian, one Rétköz Hungarian, and one Bulgarian males), cluster 6 (2 Váh valley Hungarian, two Albanian, and one Serbian males), cluster 7 (1 Hungarian, one Rétköz and one Albanian males), and cluster 8 (1 Hungarian, one Váh valley, and two Albanian males). The remaining Hungarian and other samples are scattered within the network or have shared haplotypes, like Ukrainian-Hungarian or Austrian-Bulgarian (see arrows in Figure 7).

The age of accumulated STR variation within the E1b1-M78 lineage for 183 samples is estimated to be 6.7 ± 1.3 kya (95% CI = 5.4–8.0 kya), considering the modal haplotype (see M in Figure 7) is the founder, which is in line with the arrival of Neolithic farmers in Europe (Battaglia et al., 2009).

### 3.3 Genetic structure

**FIGURE 8:**
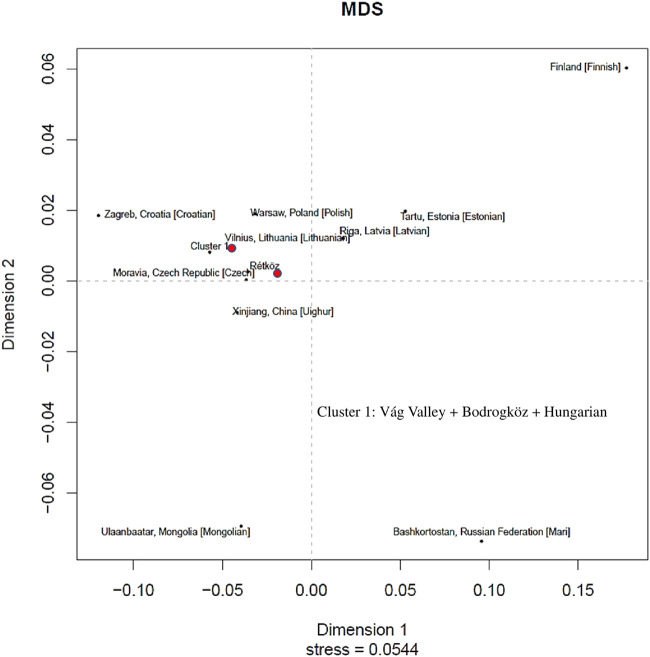
Multidimensional scaling (MDS) plot constructed on Rst genetic distances of 10 STR-based 2405 Y haplotypes of 14 populations compared (www.yhrd.org). The Rst-genetic distances and Rst *p*-values of the studied populations are presented Supplementary Table S2.

We constructed a non-metric multidimensional scaling (MDS) based on Y-chromosomal haplotypes (2,405 haplotypes) that consisted of 23 STR loci available from 14 populations (www.yhrd.org). The Rst-genetic distances and Rst *p*-values of the studied populations are presented in Supplementary Table S3. As shown in Figure 8, the Rétköz population had close Rst genetic distances (<0.05) with the Czech, Estonian, Xinjiang Uighur in China, Latvian, Lithuanian, Polish and cluster one populations. We used the relaxing MDS calculation to cluster the populations and, in this case the Rst threshold value of the indistinguishable populations was 0.01. As a result, Váh valley, Bodrogköz, and Hungarian populations formed by cluster 1. Based on the Rst genetic distances, the most distant populations with the Rétköz population were the Finnish (0.2593) and Bashkirian Mari (0.1375).

The cluster one populations showed close genetic affinities (<0.05) with most of the European populations that were compared, such as Croatian, Czech, and Polish. Interestingly, cluster one population is closely related to the Xinjiang Uighur population in China. Finnish (0.2844), Bashkirian Mari (0.1722), and Estonian (0.1181) populations, however, are genetically farthest from cluster 1.

**FIGURE 9:**
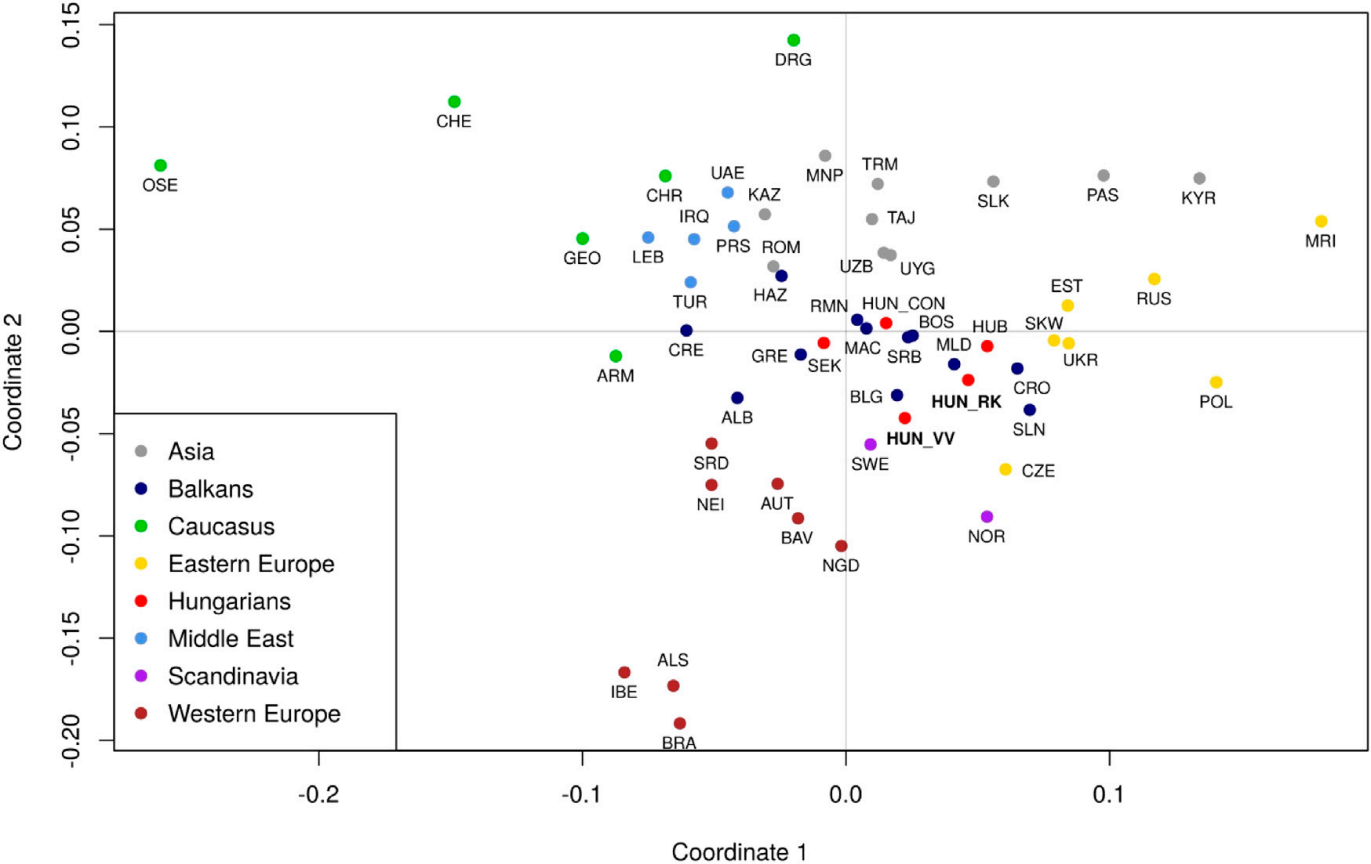
Multidimensional scaling (MDS) plot based on pairwise Fst genetic distances of 61 populations. The populations are colored based on their geographical locations. The red dots indicate Hungarian populations. Three populations are highlighted: Váh valley (HUN_VV) and Rétköz (HUN_RK) in bolded red font and Hungarian Conquerors (HUN_CON) in italic red font. Population abbreviations are defined in Supplementary Table S3. Pairwise Fst genetic distances and *p*-values between 61 populations were calculated as shown in Supplementary Table S4.

The haplogroup frequency data used for population comparisons, as well as their corresponding references are included in Supplementary Table S4. Pairwise Fst-distances and *p*-values for 61 populations, including Rétköz, Váh valley, and other Eurasian populations from published sources were calculated as shown in Supplementary Table S5 and presented in a metric MDS plot (Figure 9). Between the two studied populations, the pairwise Fst-distance was insignificant (*p* > 0.05) (Supplementary Table S5). Furthermore, Rétköz and Váh valley had insignificant Fst-values, with 5 and 11 populations, respectively. Among them, three overlapped: two Hungarian (Bodrogköz and Szeged) and a Slovenian population. Czech and Western Slovak have an insignificant Fst-distance from Rétköz, but not from Váh valley. Váh valley showed an insignificant Fst-distance from eight other populations: Bulgarian, Gagauz, Greek, Moldavian, Serbian, and three Hungarian (Csángó, Székely, and a representative Hungarian population).

The location of the studied populations on the MDS plot is consistent with the geographical distances between them (Figure 9). Populations from the same geographic region were clustered together. Hungarian populations, however, overlapped with Balkanian populations. The Rétköz and Váh valley samples were shown to be relatively far from the Hungarian Conqueror samples. A notable difference between the two populations is that the Rétköz population was genetically nearer to the Eastern European populations, whereas the Váh valley population was genetically closer to Western European populations. These observations are consistent with the populations’ geographical locations.

## 4 Discussion

### 4.1 Phylogenetic analysis

The primary objectives of this study were to create a male phylogeny of the Hungarian-speakers in Rétköz and the Váh valley and to compare them with other Eurasian populations, with aDNA samples from their probable ancestral geographical origin, and with previously studied populations.

To examine the genetic variation within the Hungarian groups, we used evolutionarily stable binary markers (SNPs) to define the haplogroup of each Y-chromosome, and then examined the STR-defined variation within each haplogroup. The established geographical specificity of Y haplogroups means that the haplogroups observed in the Hungarian-speakers may be attributed to their geographical origins or may be related to the Hungarian Conquerors. These conclusions, however, remain uncertain.

The overall pattern of haplogroup distributions in the studied populations was similar, but haplogroups R1a-Z93, N1c-L1034, N1c-VL29, N1c-Z1936, and Q-M242 appeared only in the Rétköz population (Table 1). Haplogroups G2a-L156 and R1b-M343/P25 (L23) were observed more frequently in the Rétköz population. Both lineages range predominantly across the Caucasus and Asia. As such we focused on the genetic history of these haplogroups.

#### 4.1.1 Median-joining network of 121 R1a-Z93 haplotypes

Underhill et al. state that the paragroup R1a-Z93 was most common in the South Siberian Altai region but that it also occurred in Kyrgyzstan and, in all Iranian populations. R1a-Z2125 occurred at highest frequencies in Kyrgyzstan and among Afghan Pashtuns. Additionally, the R1a-Z93 haplogroup was also common in Afghan (Tajik) and Caucasian ethnic groups (Tajik). As such, we included the populations (Z93 haplotypes) from the regions analyzed by other researchers (Underhill et al., 2015), ancient Hungarian, Xiongnu, and Avar samples (Olasz et al., 2018; Csáky et al., 2020; Fóthi et al., 2020; Keyser et al., 2021) in the network analysis.

It is surprising that the present-day Hungarian, ancient Hungarian and Xiongnu samples are located adjacent to each other on this branch, indicating that 1-1 mutation step separates them. Keyser et al., 2021 demonstrated that ancient Xiongnu (TUK45, TUK04, TUK25 and TUK09A) samples belonged to haplogroups R1a-Z93 (Z2125), which are also included in our network. These samples clustered with our Hungarian aDNA (II54) and three present-day Hungarian samples, including Rétköz 40 (R40), in the branch mentioned above (see Figure 2). Furthermore, a Hungarian aDNA haplotype (Nagykörös Gr2) shared the same haplotype as the Xiongnu TUK09A sample (cluster one in Figure 2.). Likewise, a modern Hungarian sample shared the same haplotype as the Xiongnu TUK25 sample (cluster three in Figure 2).

aDNA studies showed that the Hungarian King Béla III, and another sample (II54) from Royal Basillica belonged to haplogroup R1a-Z93 (Olasz et al., 2018), and two R1a-Z93 samples were found among Hungarian Conqueror population as well (Fóthi et al., 2020). Additionally, some Xiongnu R1a-Z93 haplotypes matched those of the Hungarian Conquerors (Keyser et al., 2021). These observations confirm a genetic relationship between Hungarian Conqueror and the ancient Xiongnu people, suggesting descent from a shared common ancestor. These observations also reflect that the descendants of the Xiongnu people and the ancient Hungarians are found among modern Hungarian populations, indicating the presence of a common genetic trace (i.e., genetic continuity).

#### 4.1.2 Median-joining network of 153 N-M46 haplotypes

The Y-chromosomal haplogroup N-M231 is widespread from Scandinavia to the Kamchatka in North Eurasia and is the most frequent single haplogroup in Siberia (Karafet et al., 2008; Rootsi et al., 2012). Based on the geographic distribution of the most basal N*-M231, its lineage most likely originated in Southeast Asia, whereas its most widespread subgroup is N-M46 (Rootsi et al., 2012). Other authors consider Southern Siberia as its geographical origin (Derenko et al., 2007).

The Hungarian Y-chromosomal gene pool has only a small percentage of N-M46 (1%; Tat = M46) and has a distribution typical of East-Central Europe (Völgyi et al., 2009). However, its incidence is higher among the Hungarian-speaking Bodrogköz (6.2%) and Székely (6.3%) (Transylvania, Romania) populations (Biró et al., 2015; Pamjav et al., 2017). Based on Hungarian aDNA studies, N-M46 is detected at a higher frequency in Hungarian Conquerors (Neparaczki et al., 2019; Fóthi et al., 2020). These results support genetic continuity between the ancient Hungarian and the present-day Hungarian populations.

The Rétköz N-M46 sample 1 (R1) from this study and four other Hungarian samples belong to haplotype cluster one and share 31 haplotypes with 8 Eurasian populations (Hungarian, Mongolian, Southern Mansi, Avar, Northern Mansi, Finnish, Bashkirian, and Khanty), indicating they may all share a common genetic history (Figure 3). The second Rétköz (R19) N-M46 sample shared a haplotype with the general Hungarian population (see three in Figure 3.). The third Rétköz (R4) haplotype does not share a haplotype and instead clusters with Finnish haplotypes. This is consistent with the N-Z1936 subgroup, as this SNP is common in the Finnish population. Other modern Hungarian N-M46 samples, including Hungarian aDNA (aH: Őrkút Gr50), are located on the top or the right side of the network, along with Bashkirian, Finnish, and samples originating in the Ural Region (Southern and Northern Mansi, Khanty). Three Hungarian males share a haplotype with the Finnish, Northern Mansi, and Bashkirian samples, suggesting a shared ancestor (see the black arrows). One aDNA Xiongnu (X: TUK30A) sample from Mongolia clusters with Mongolian samples on the left side of the network, indicating a genetic relationship between these groups.

#### 4.1.3 Median-joining network of 153 R1b-M343 haplotypes

The most frequent Western European lineage, haplogroup R1b-M269, was originally thought to have originated in the Palaeolithic. Recent analysis, however, suggests a Neolithic origin (Batini et al., 2017). Most R1b-M412 chromosomes belong to Western European males, but another subgroup, R1b-L23, is commonly referred to as “Eastern European R1b”. Its frequency among Turkish, Caucasian, and some SE European and Circum-Uralic populations is about 10% (Myres et al., 2011).

Our network analysis showed that the R1b*-M343 (xM412) haplotypes are divided into two subclades: R1b-L23 (xM412) and R1b-M73. The Rétköz and other Hungarian haplotypes cluster with the L23 samples included in the study and share haplotypes with Hungarian, Abkhazian, and Avar males from the Caucasus, suggesting a genetic link between them (see Figure 4). These Avars are unlikely the same as the Pannonian Avars in the Carpathian Basin because present-day examples live in the Northeastern Caucasus, primarily in Dagestan, Kalmykia, and Chechnya in Russia. The earliest mention of the Avars in European history is by Priscus, who reported in 463 C E that a joint delegation of Saragurs, Urogs, and Unogurs requested an alliance with Byzantium. The delegation claimed that in 461 CE, their peoples were displaced by the Sabirs due to pressure from the Avars (Priscus, 463 CE). The German researcher Karl Heinrich Menges stated that these Avars have nothing to do linguistically with the Proto-Mongolian Avars of the Great Migrations, but as some of the latter may have taken refuge in this region, their name has become the common name of the Avars of present-day Dagestan, who have no name of their own (Menges, 2011).

The haplotype analysis revealed a genetic relationship between the contemporary Hungarian-speakers and Caucasian populations (Avars, Abkhazians, and Ossetians) included in this study. It should be noted, however, that the peoples of East-Central Europe received Central-Inner Asian and Caucasian genes before and after the ancient Hungarians settled in the Carpathian Basin (pre-Hungarians: Sarmatian-Alans, Huns, Avars, and Onogur-Bulgars; post-Hungarians: Pechenegs, Jassic peoples, and Cumans) (Lipták 1979). Based on aDNA studies, haplogroup R1b-L23 was found in Hungarian Conquerors (Neparáczki et al., 2019; Fóthi et al., 2020), supporting the genetic footprint observed in our results, but it was not possible to determine when and where this genetic trace was introduced into the gene pool of the ancient Hungarians.

#### 4.1.4 Median -joining network of 167 Q-M242 haplotypes

The human Y-chromosome haplogroup Q-M242 likely originated in Central Asia and Southern Siberia 15–25 kya (Karafet et al., 2008), and then subsequently diffused eastward, westward, and southward (Di et al., 2013; Huang et al., 2018). Haplogroup Q-M242 reaches its highest frequencies in Siberia, particularly in Kets (90–94%) and Selkups (66–71%) but is rarely found in Western, Southern, and Southeastern Asia (Di et al., 2013; Huang et al., 2018). Subclade Q-M120 occurs in Eastern Asia and migrated from north to south with ancestors of the Han Chinese during the Neolithic period (Gayden et al., 2007; Zhao et al., 2015). Subclades Q-M25 and Q-M346 spread widely in Eurasia. Q-M25 reaches its highest frequency among the Turkmen (34–43%) and spread from Central Asia into Western Asia and Hungary, whereas Q-M346 appears in most parts of Eurasia (e.g., Central, Western, and Southern Asia), as well as the Comoros Islands of Africa (Sengupta et al., 2006; Malyarchuk et al., 2011; Huang et al., 2018). Subclade Q-M3 is present only in Indigenous Americans (Grugni et al., 2012).

Based on the network analysis of haplogroup Q-M242, is that Hungarian-speaking populations living in more isolated areas (no admixture), such as Csángó and Székely males in Transylvania, Romania, clustered on the same branch as the ancient Xiongnu people lived in Inner Asia about 2000 years ago, suggesting these males are genetically related (see Figure 5). An Uzbek sample from Khwarezm (Biró et al., 2015) is also on this branch, in addition to this study’s R80 sample, which shares the same haplotype as an Uzbek sample from China (Huang et al., 2018), implying a common genetic trace. Neparáczki et al., 2019 reported that, in Hungary, haplogroups Q-M25 and Q-F1096 (xM25) were found in the Hunnic period (fifth century CE) and the Hungarian Conquest period (9th-11th centuries CE), respectively. They state that although Q-M25 is rare in Europe its highest frequency is among Hungarian speaking Székely population in Transylvania, Romania. Furthermore, the authors noted that ancient samples with haplogroup Q1a2-M25 are known from the Bronze Age Okunevo and Karasuk cultures, as well as from Middle Age Tian Shan Huns and Hunnic-Sarmatians, suggesting that this lineage may be of Hunnic origin in Europe. This is confirmed by the Hun/1 sample, derived from Transylvania (Neparáczki et al., 2019). These observations also confirm the genetic relationship between contemporary Hungarians (Székely and Csángó) and the ancient Xiongnu people of Inner Asia that we found in our study.

The remaining two Rétköz Q samples (R101 and R58) cluster with Central (Uzbek, Tajik, and Turkmen) and Inner Asian (Mongolian) samples in a subbranch of the main branch, which indicates that these samples are distant from the Hungarian-Xiongnu subbranch but that they nevertheless originate from a common branch (Figure 5).

Another interesting observation is that three Hungarian males on the Hungarian-Xiongnu branch (Figure 5) form a cluster with Balkarian males in another network (see Results, data not shown). The Balkars identify as a Turkic people and speak the same language as the Karachays from Karachay-Cherkessia. Balkars and Karachay are sometimes referred to as a single ethnicity (Джантуева 2010). Our comparative phylogenetic study of folk music and genetics, indicate that the Volga–Sicilian–Turkish–Karachay–Hungarian–Finnish–Dakota and the Chinese–Mongol–Volga–Sicilian–Turkish–Karachay–Hungarian cultures have the largest sets of common melody types, suggesting the existence of a common “parent language” from which their music evolved. The results may also show that the populations that incorporate this musical style into their culture have common genetic roots, in particular that the development of this musical culture in their early history may be attributed to their common genetic ancestors (Pamjav et al., 2012). In this study, the similar melodies in Hungarian and Karachay folk music may suggest their common genetic traces. Unfortunately, however, comprehensive genetic studies on Balkars/Karachays are not available.

#### 4.1.5 Median -joining network of 189 G2a-L156 haplotypes

Haplogroup G isassociated with the spread of agriculture, particularly in Europe. Haplogroup G was first discovered in Europe and Georgia (Semino et al., 2000) and was later detected in Caucasian and Hungarian populations (Nasidze et al., 2004; Völgyi et al., 2009). The frequency of haplogroup G2a-P15 (L156) is about 4% in the general Hungarian population (Völgyi et al., 2009), 6% in the Hungarian-speaking Csángó population (Transylvania, Romania), and 4% in the Hungarian-speaking Székely population (Transylvania, Romania) (Bíró et al., 2015). As the frequency of the G haplogroup is low among Hungarian-speakers, only the L156 SNP was tested from the downstream SNPs, which is phylogenetically equivalent to SNP P287 marker (www.phylotree.org/Y/tree/G.htm). Rootsi et al. analyzed 113 Hungarian males, of which 2 belonged to haplogroup G-M201 (1.8%) and to subgroups G-L497 (0.9%) and G-M406 (0.9%). These authors showed that SNP P303 defines the most frequent and widespread subhaplogroup G, whereas P303-related L497 lineages occur in Europe, where they likely originated. The highest frequency of G2a-P303 is detected in populations from the Caucasus, specifically among South Caucasian Abkhazians (24%), Northwest Caucasian Adyghe (39.7%), and Cherkessians (36.5%) (Rootsi et al., 2012). Another frequent subclade is M406, which is the sister clade of P303. The G2a-M406 has a peak frequency in the Mediterranean and Central Anatolian (6–7%) populations, as well as in Greek (4%) and Italian (3%) populations. It is not detected in many other regions with high P303 frequency (Rootsi et al., 2012). The G2a-P16 (P18) lineage is specific to the Caucasus, accounts for a third of the Caucasian male gene pool and has a high frequency in the Southern and Northwestern Caucasus, with the highest frequency among North Ossetians (63.6%). Outside the Caucasus (Anatolia, Armenia, Russia, and Spain), the P16 lineage is either present at less than 1% or is absent (Rootsi et al., 2012).

Based on our network analysis, four subclades can be distinguished as L497, P303, M406, and P18 (under P16), as the samples with known haplogroups in the network are clustered on the same branches. Hungarians are included in L497, P303, and M406 subclades, except for P18 (1 Csángo sample), indicating that the gene flow from the P18 subclade of the Caucasus has been negligible. We included nine samples from the Rétköz and Váh valley populations, each of which falls into these three subgroups. The results indicate a close genetic relationship between the Hungarian-Balkar/Karachay and Hungarian-Avar males, because they share common haplotypes (cluster 1–3) or cluster in similar haplotypes (P303, L97, and M406). Thus, these observations likely suggest a common genetic origin, rather than coicidence.

The results of the aDNA studies in the Carpathian Basin show the haplogroup G2a-L156. One ancient Avar and four Hungarian Conqueror samples belong to the subgroup G2a-L293, two Hungarian Conqueror samples belong to haplogroup G2a-U1, and one ancient Hungarian sample belongs to haplogroup G2a-L30 (Neparáczki et al., 2019; Fóthi et al., 2020). SNPs L293 and L30 are downstream of L156 but upstream of M406. U1 and L497 are sister clades of P303 under M406 SNP (http://www.phylotree.org/Y/tree/G.htm). This indicates that SNPs L293 and L30 are older than SNPs M406 and P303, whereas SNPs U1 and L497 are the youngest.

As such, the modern and ancient DNA results support the case for a common genetic origin of the Hungarian and Caucasion populations.

#### 4.1.6 Median -joining network of 183 E1b1-M78 haplotypes

Haplogroup E, defined by mutation M40 (M96, P29), is the most common human Y-chromosome clade in Africa (www.phylotree.org/tree/E/htm **)**. From downstream SNPs of haplogroup E, the E1b1-M35 mutation and M78 below it are two of the most frequent markers in European males. E-V13, a single clade within E, highlights a series of expansions duirng the Bronze Age in Southern Europe (Cruciani et al., 2007). All European M78 chromosomes belong to subclade V13, as well (www.phylotree.org/tree/E/htm). The highest frequency values for the M78 lineage are detected in populations from the Balkans, such as Albanians (32.29%), Bulgarians (16.67%), Macedonians (18.18%), continental Greeks (19.05%), and Southern Italians (13.07%) (Cruciani et al., 2007). The frequency of haplogroup E1b1-M78 is about 4.2% in the general Hungarian population (Völgyi et al., 2009) and 9.43% in the Hungarians analyzed by Cruciani et al. (2007). However, its presence is negligible in Caucasian populations, like the Lezghins, Ossets-Digor, Ossets-Iron, Abkhazians, Shapsugs, and Circassians (Balanovsky et al., 2011), and it is very rare in Central and Inner Asia (Biró et al., 2015).

As shown in Figure 7, the E1b1-M78 chromosomes of Hungarian-speaking populations originated in the Balkans and were introduced into Hungarian gene pool. Their location on the outer edge of the network, indicating many mutational steps, suggests that the M78 chromosomes in the Váh valley population appear to have separated from the common European ancestor earlier than those of the Rétköz population.

In aDNA studies, E1b1-M78 was observed among the Middle and Late period Avar (650–710 CE), as well as among the Hungarian Conquerors (895-mid *X*th century) (Neparáczki et al., 2019). However, the absence of STR precludes its inclusion in the network analysis.

Based on the network topologies, the most diverse haplogroups are Q-M242, G2a-L156 and R1b-L23, which can be seen from the pattern of the networks, that is, the number of unique haplotypes of a male within the network is significant as well as the age of the accumulated STR variation (TMRCA) also supports it. The divergence times of haplogroups N-M46 and R1a-Z93 are almost the same (TMRCA) and within the network there are many clusters where several males share the same haplotype. Haplogroup E-M78 is the youngest, as the number of unique haplotypes is less than in the other networks.

### 4.2 Genetic structure

Based on Fst analysis, the Váh valley population shows a stronger genetic similarity to Balkan populations than does the Rétköz population. This is primarily due to the relatively high E and I2a haplogroup frequencies. Both haplogroups are common in Balkan populations but are less frequently found in Eastern Europeans (Regueiro et al., 2012; Doğan et al., 2016). The Rst-based MDS plot supports this observation, as the Váh valley popular was closer to a Balkan population (Croatia), whereas the nearest population to Rétköz is a Central European population (Czech). A further difference between the two studied Hungarian populations can be concluded from the Fst-based MDS plot. It shows that the Váh valley, which is at the western part of the Hungarian-Slovakian contact zone, is genetically closer to the Western Europeans and that Rétköz is at the eastern part of that zone and is therefore genetically closer to the Eastern Europeans. This study did not detect a genetic linkage between the Váh valley and the Hungarian Conquest period populations, and the results also reveal the Váh valley’s relative isolation from neighboring populations.

In summary, the results obtained by us, which show that the genetic relationships between modern Hungarians, Hungarian Conquerors, Asian Huns (Xiongnu) and ancient Avars are continuous, are fully supported by the results of the whole genome sequencing data performed by Hungarian researchers (Maróthi et al., 2022).

## 5 Conclusion

The genetic composition of the Rétköz (Hungary) and Váh valley (Slovakia) populations indicate different histories. In the Rétköz population, the paternal lineages that were also found in the Hungarian Conquerors, such as haplogroups R1a-Z93, N-M46, Q-M242, R1b-L23, and G2a-L156, were better preserved. The genetic composition of the Váh valley population is similar to that of the surrounding Indo-European populations.

The Hungarian males shared common haplotypes with ancient Xiongnu, ancient Avar, Caucasian Avar, Abkhazian, Balkarian, and Circassian males within haplogroups R1a-Z93, N1c-M46, and R1b-L23, suggesting a common genetic footprint. Additionally, Hungarians cluster on a common branch with the ancient Asian Huns (Xiongnu), ancient Avars, and several modern Caucasian populations (Avar, Ossetian, Balkars) within the haplogroups R1a-Z93, R1b-L23, and Q-M242, implying a close genetic relationship.

Further studies are needed to clarify if the common genetic footprints were acquired directly or indirectly. Comprehensive studies from European, Central Asian, and Caucasian populations should be conducted for the haplogroups using more downstream SNPs and NGS sequencing to learn more about the origins, expansion, and ethno-linguistic affiliations of the populations.

## Data Availability

The data presented in the study can be found in the YHRD respository, accession numbers: YA004754 and YA0047555.
